# Efficient Adsorption on Benzoyl and Stearoyl Cellulose to Remove Phenanthrene and Pyrene from Aqueous Solution

**DOI:** 10.3390/polym10091042

**Published:** 2018-09-19

**Authors:** Yohan Kim, Daham Jeong, Kyeong Hui Park, Jae-Hyuk Yu, Seunho Jung

**Affiliations:** 1 Department of Systems Biotechnology, Microbial Carbohydrate Resource Bank (MCRB), Center for Biotechnology Research in UBITA (CBRU), Konkuk University, Seoul 05029, Korea; shsks1@hanmail.net (Y.K.); amir@konkuk.ac.kr (D.J.); kyeonghee17@naver.com (K.H.P.); 2 Departments of Bacteriology and Genetics, University of Wisconsin-Madison, Madison, WI 53706, USA; jyu1@wisc.edu

**Keywords:** adsorption, phenanthrene, pyrene, benzoyl cellulose, stearoyl cellulose

## Abstract

Benzoyl and stearoyl acid grafted cellulose were synthesized by a simple chemical grafting method. Using these as chemical adsorbents, polycyclic aromatic hydrocarbons (PAHs), like pyrene and phenanthrene, were effectively removed from aqueous solution. The structural and morphological properties of the synthesized adsorbents were determined through X-ray diffraction analysis (XRD), thermal gravimetric analysis (TGA), Fourier transform infrared (FT-IR), FE-SEM, and NMR analyses. Through this method, it was confirmed that benzoyl and stearoyl acid were successfully grafted onto the surface of cellulose. The 5 mg of stearoyl grafted cellulose (St–Cell) remove 96.94% pyrene and 97.61% phenanthrene as compared to unmodified cellulose, which adsorbed 1.46% pyrene and 2.99% phenanthrene from 0.08 ppm pyrene and 0.8 ppm phenanthrene aqueous solution, suggesting that those results show a very efficient adsorption performance as compared to the unmodified cellulose.

## 1. Introduction

Industrial and agricultural contaminants, the number of which has dramatically increased due to worldwide development, are the most serious pollutants globally. Water resources can be contaminated with organic compounds, which are highly hazardous in every aspect. It has been reported that organic molecules in water from manufacturing activities, such as dyes, agrichemicals, and combustion byproducts, are not only a global concern owing to their long-term potential risk to ecosystems, but also have an effect on human health due to their high toxicity and low biodegradability [1]. Therefore, numerous countries regard water resource policy as a government priority project for decades. Activated carbon is a common adsorption material for the harmful substance, but it is expensive and the regeneration is difficult [2]. Recently, research on environmentally friendly materials for the removal of these pollutants have been intensified. Candidate materials have been reported in various forms such as hydrogel, nanocomposite, and membrane. These various platform materials were prepared by chemical modification with bio-based materials such as alginate [3], chitosan, cellulose [4], lignin [5], gelatin [6], and soy [7], thereby controlling harmful substances.

Among the organic products of incomplete combustion found in coal and in tar deposits, Polycyclic Aromatic Hydrocarbons (PAHs) are some of the most harmful carcinogenic substances [8,9]. PAHs are composed of multiple aromatic rings, which renders them stable and bioavailable from months up to several years. Therefore, many researchers have attempted, through various treatments, to efficiently remove PAHs from water. However, it is difficult to completely convert PAHs to nontoxic molecules, such as CO_2_ and H_2_O, by chemical treatments [10]. The highly effective treatments, such as ozonation, high energy electron beam irradiation, and catalytic combustion, are high-cost methods, and so they are not extensively employed [11,12]. Among the diverse treatment techniques that have been applied by many researchers, sorption technology is the most common method to remove organic pollutants in waste water. This method is advantageous due to its simple and efficient methodology, which can be applied at any scale. Therefore, adsorption techniques can be used to remove both hazardous and less soluble organic molecules, such as PAHs, from water. However, the effectiveness of this method can be restricted because the sorption capacity can be affected by the chemical structure of the material and by its characteristics, such as porosity, specific surface area, swelling, and diffusivity. Many studies have been conducted to prove the sorption efficiency of various candidate materials as an adsorbent to remove PAHs. Amongst these candidate materials, cellulose has some advantages as an adequate adsorbent [13].

Cellulose is inexpensive and environmentally friendly because it is the most abundant naturally made polymer on the earth. Cellulose derivatives have been applied to the filtration process as these materials can trap organic and inorganic pollutants [14,15,16]. However, the adsorption ability of these naturally made products varies due to its dependence on the chemical environment. In order to complement the insufficient adsorption capacity, modification of cellulose has been carried out in numerous studies for decades [17,18,19]. Because the chemical modification on the hydroxyl group of the cellulose backbone changes the performance, as well as its properties, this method has been used to alter certain features of cellulose, such as hydrophobic or hydrophilic properties and elasticity [20]. Among many chemical cellulose modification methods, such as esterification, halogenation, oxidation, and etherification, determining adequate chemical reaction conditions is essential. Acylation of cellulose is a rapid esterification reaction with a high yield [21,22,23]. Particularly, the desired modified cellulose can be obtained by treating cellulose with other organic polymers containing long chains or specific functional groups. These hydrocarbon moieties or functional groups on the cellulose are then able to interact with organic molecules in aqueous solutions.

In this study, the modified cellulose that was successfully prepared by esterification reaction with stearoyl chloride and benzoyl chloride is presented. The properties and surface morphology of the synthesized material were determined and characterized by various modern characterization techniques. Also, the sorption efficiency of stearoyl and benzoyl cellulose, as measured by spectroscopic analysis, was shown as the removal percentages of phenanthrene and pyrene from aqueous solution. From the results of this study, the direction and efficiency of the experiment were determined and discussed in comparison with previous studies.

## 2. Materials and Methods

### 2.1. Materials

A cellulose microcrystalline powder (20 µm) was purchased from Sigma-Aldrich (St. Louis, MO, USA). The cellulose was dried in a vacuum oven at 60 °C for 12 h to remove moisture before further reaction. Benzoyl chloride (99.0%), stearoyl chloride (97.0%), 4-dimethylamino pyridine (DMAP) (99.0%), lithium chloride (LiCl) (99.0%), anhydrous 4-*N*,*N*-dimethylacetamide (DMAc) (99.8%), phenanthrene (98.0%), and pyrene (98.0%) were purchased from Sigma-Aldrich.

### 2.2. Synthesis of Benzoyl Cellulose (Bz–Cell) and Stearoyl Cellulose (St–Cell)

Bz–Cell and St–cell were prepared based on previously described methods with some modification [24]. The whole reaction performed under nitrogen atmosphere. A cellulose microcrystalline powder (1 g) was dissolved in 40 mL of DMAc containing 8 wt % LiCl at 100 °C for 4 h. Then, benzoyl chloride (4.336 g) or stearoyl chloride (9.345 g) was added and DMAP (0.376 g) was used for catalyst. The reaction was constantly stirred at 100 °C for more than 12 h. The reaction mixture was precipitated with deionized water, and the resulting solid was filtered out and washed repeatedly using chloroform/methanol. The obtained Bz–Cell or St–Cell was kept in a vacuum oven at 80 °C for 24 h and then utilized for further study. The yields of Bz–Cell and St–Cell were 93% and 99%, respectively. The synthesized compounds (Bz–Cell and St–Cell) were deposited in the Microbial Carbohydrate Resource Bank (MCRB) at Konkuk University, Korea.

### 2.3. NMR Spectroscopy

NMR spectra was recorded on a Bruker Avance 600 MHz spectrometer (A Bruker Avance spectrometer, Bruker, Karlsruhe, Germany) for ^1^H NMR and ^13^C NMR experiments. The chloroform-d was used for dissolution of the samples at 25 °C for measurement.

### 2.4. Fourier Transform Infrared (FT-IR) Spectroscopy

Fourier transform infrared (FT-IR) spectra were conducted with a Bruker IFS-66/S spectrometer (Bruker, Karlsruhe, Germany) while using KBr pellets as support in the scanning range of 650–4000 cm^−1^.

### 2.5. Field Emission Scanning Electron Microscopy (FE-SEM)

Hitachi S-4700, which is manufactured by Hitachi High-Technologies Corporation (Tokyo, Japan) was used for field emission scanning electron microscopy (FE-SEM). Double-sided adhesive carbon tape was used to fix the samples on a brass stub. The powder samples were coated on the surface in a thin layer of gold. The images were photographed at an excitation voltage of 10 kV.

### 2.6. X-ray Diffraction Analysis (XRD)

X-ray diffraction analysis (XRD) was performed on a Bruker D8 DISCOVER diffractometer (Bruker, Karlsruhe, Germany) while using Cu-Kα radiation. It recorded XRD patterns by analyzing diffractions at a 2θ angle values between 3° and 43° in 1° min^−1^ increments and a recording time.

### 2.7. Thermal Gravimetric Analysis (TGA)

Thermogravimetric analysis (TGA) curves were obtained with TA Instruments (Q20, New Castle, DE, USA). For TGA measurements, it recorded the percentage weight loss of the dried sample under a flowing nitrogen atmosphere from 25 to 600 °C and a heating rate of 10 °C min^−1^.

### 2.8. Adsorption of Phenanthrene and Pyrene

We chose phenanthrene and pyrene as PAHs representative. Due to the low water solubility of phenanthrene and pyrene (1.1 and 0.13 mg/L), the both PAHs were dissolved in methanol high concentrations. To avoid co-solvent effects, the solutions were diluted with deionized water to make 0.1% of final methanol concentrations [25]. Each of Bz–Cell and St–Cell were placed into screw cap vials with 0.8 ppm of phenanthrene or 0.08 ppm of pyrene solution, respectively, and the mixture solutions were stirred and equilibrated at 25 °C for 24 h. The solutions containing phenanthrene and pyrene were filtered and then measured by a spectrofluorophotometer with ex/em wavelengths of phenanthrene (ex/em = 248 nm/364 nm) and pyrene (ex/em = 335 nm/392 nm). The obtained emission spectra were shown in the each range of phenanthrene (300–450 nm) and pyrene (300–450 nm). The excitation and emission slits was 3.0 and 1.5 nm widths, respectively. The removal efficiency of PAHs could be calculated, as follows:

(1) $$\mathrm{Removal}\;\mathrm{efficiency}=\frac{C_{0}-C_{e}}{C_{0}}\times 100\%$$

where *C*_0_ is the initial concentration of PAHs, and *C*_e_ is the concentration of PAHs after adsorption process.

### 2.9. Recyclability of Bz–Cell and St–Cell

After completion of the adsorption experiment, the Bz–Cell and St–Cell were collected by centrifugation, washed with methanol, and then lyophilized. The recovered Bz–Cell and St–Cell were then reused for the next run. We also measured fluorescence of methanol, which was used in washing step for each adsorbent to check the desorption of both phenanthrene and pyrene (Figure S1, Supplementary Materials).

## 3. Results

### 3.1. Characterization of Bz–Cell and St–Cell

**Scheme 1:**
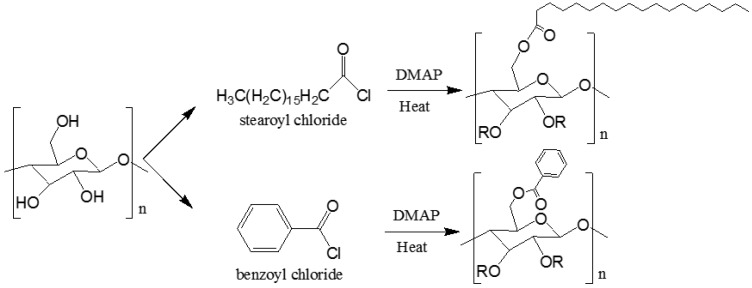
Stearoyl cellulose (St–Cell) and benzoyl cellulose (Bz–Cell) synthesis method.

**Figure 1:**
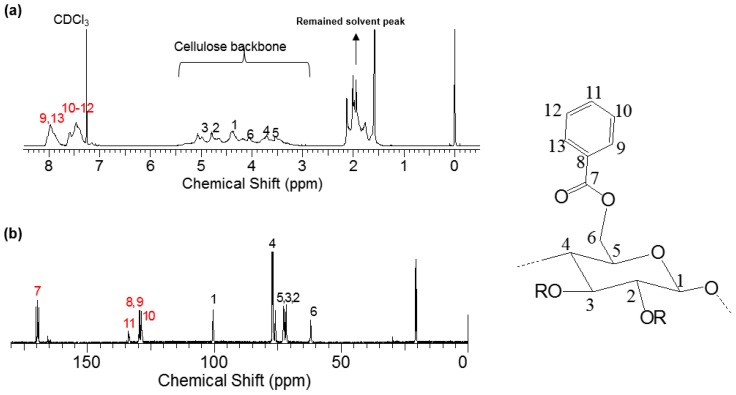
^1^H NMR spectrum (**a**) and ^13^C NMR spectrum (**b**) of the Bz–Cell.

**Figure 2:**
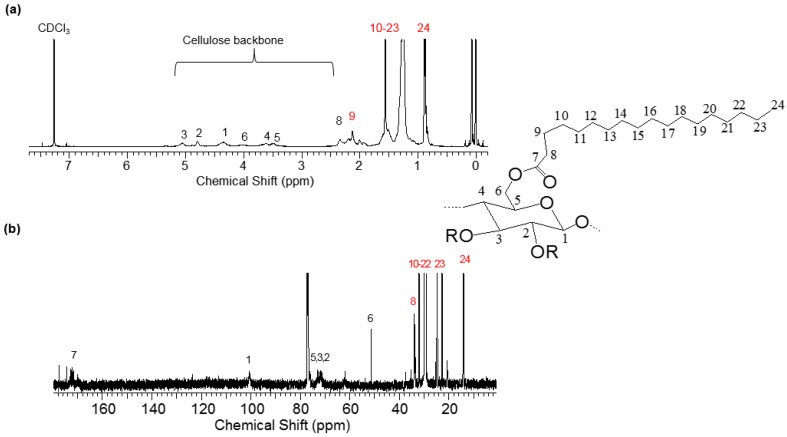
^1^H NMR spectrum (**a**) and ^13^C NMR spectrum (**b**) of the St–Cell.

The Bz–Cell and St–Cell were synthesized via the reaction of benzoyl chloride and stearoyl chloride with dimethylacetamide (DMAP), respectively (Scheme 1). Cellulose was dissolved by DMAc and LiCl. Strong hydrogen bonds are formed between the hydroxyl protons of cellulose and the Cl^−^ ion with solvation of the Li^+^ ions by free DMAc molecules. Once nucleophile (DMAP) donated an electron pair to acyl carbons of the substituents, such as benzoyl chloride and stearoyl chloride, it would be able to react with hydroxyl groups on 2, 3, and 6 position of cellulose. In the ^1^H NMR spectrum of the Bz–Cell, cellulose backbone protons appear in the range of 3.00–5.00 ppm, and the benzoyl ring protons are visible at 7.49 ppm (10–12) and 7.9 ppm (9, 13). The peaks of remained solvent appeared at around 1.5–2.0 ppm. In fact, some solvents were difficult to remove by washing because the Bz–Cell was aggregated and kept hydroxyl groups of cellulose making strong binding with DMAc molecules [26]. It has been reported that when synthesizing benzoyl cellulose in the DMAc/LiCl system with DMAP, remained solvent peak can be appeared in the ^1^H NMR spectrum despite a sufficient washing process (Figure 1a) [27]. In the ^13^C NMR downstream spectrum, the peaks of the benzoyl ring carbons were distributed over 125 ppm (Figure 1b). In the same way, cellulose backbone protons of the Bz–Cell were in the same ^1^H NMR spectrum range (Figure 1a). The ^1^H NMR and ^13^C NMR spectra of the St–Cell are shown in Figure 2. The characteristic peaks due to stearoyl carbon chain protons are observed, ranging from 0.86 to 2.18 ppm (9–24) (Figure 2a). In the ^13^C NMR upstream spectrum, the peaks of stearoyl carbons were distributed in the region under 35 ppm (Figure 2b).

### 3.2. FT-IR Spectroscopic Analysis

**Figure 3:**
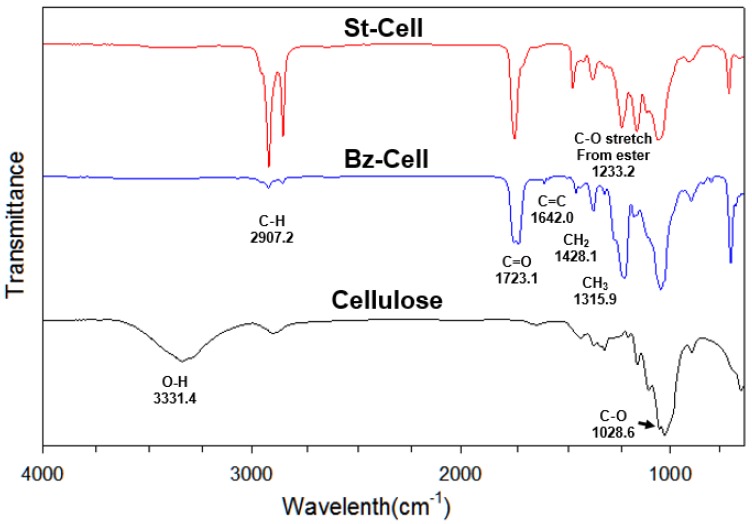
Fourier transform infrared (FT-IR) spectra of cellulose (black); Bz–Cell (blue); and St–Cell (red).

A broad absorption band at 3331.4 cm^−1^, which corresponds to hydroxyl group valence vibrations in cellulose, was observed in the pure cellulose. In the case of the Bz–Cell and St–Cell, this band was not observed because of the esterification reaction of pure cellulose, which modified with benzoyl and stearoyl chloride on an OH-group of the pure cellulose (Figure 3). The other characteristic band, representing the ester C=O bond (1723.1, 1743.3 cm^−1^) was also observed due to the esterification of the Bz–Cell and St–Cell (Table 1). The appearance of a C=C band at 1642.0 cm^−1^ in the Bz–Cell suggests the presence of benzoyl groups in the Bz–Cell. The vibrations of C–H bonds in stearoyl moiety in the St–Cell corresponded to the appearance of strong bands around 2917.8 cm^−1^ supporting that the esterification reaction successfully occurred in the synthesis of St–Cell.

From the FT-IR spectra of the modified cellulose Bz–Cell and St–Cell, the number of hydrogen bonds decreased due to the low solubility from the substitution of hydroxyl groups with hydrophobic substituent benzoyl chloride and stearoyl chloride, respectively, on the cellulose backbone. Therefore, a substantial reduction was observed in the –OH detecting area and the characteristic ester C=O absorption band at 1723.1 and 1743.3 cm^−1^ was observed. Even though the hydroxyl groups on the cellulose backbone were not fully substituted, all the peaks belonging to the benzoyl and stearoyl groups were absent in pure cellulose, and, therefore, the cellulose was modified by the esterification reaction and thus, Bz–Cell and St–Cell were successfully synthesized.

### 3.3. Thermal Gravimetric Analysis (TGA)

**Figure 4:**
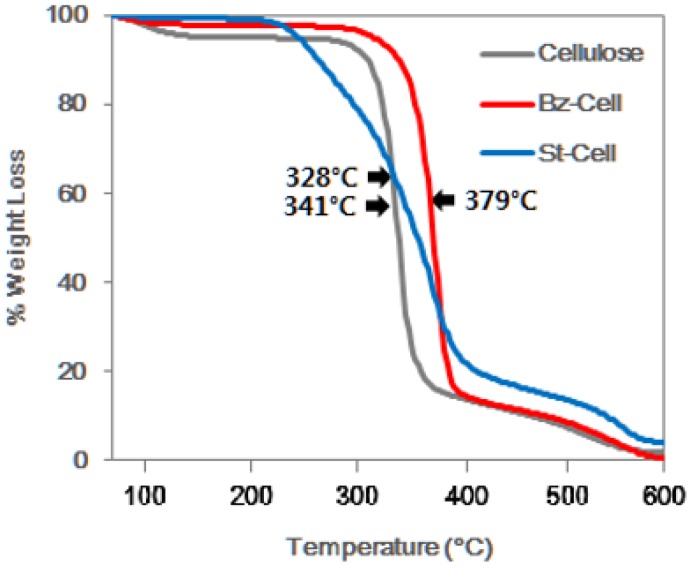
Thermal Gravimetric Analysis (TGA) analysis of cellulose (gray); Bz-Cell (red); and St-Cell (blue).

Thermogravimetric analysis of pure cellulose, Bz–Cell, and St–Cell was carried out to monitor the effect of the benzoyl and stearoyl groups on the thermogravimetric properties of the prepared materials. The decomposition temperature (°C) of pure cellulose, and the benzoyl and stearoyl cellulose materials, against weight loss (%), are plotted in Figure 4. The increase in *T*_g_ value reflects the modification of benzoyl and stearoyl on cellulose and the stearoyl chain interactions between them, which enhances the chain packing of the material. In particular, pure cellulose showed a *T*_g_ at 328 °C, whereas benzoyl cellulose and stearoyl cellulose had a *T*_g_ of 379 and 341 °C, respectively (Table 2). These results suggest that pure cellulose was modified with benzoyl and stearoyl groups along its backbone, as evidenced by the higher *T*_g_ of the Bz–Cell and St–Cell.

### 3.4. XRD Analysis

**Figure 5:**
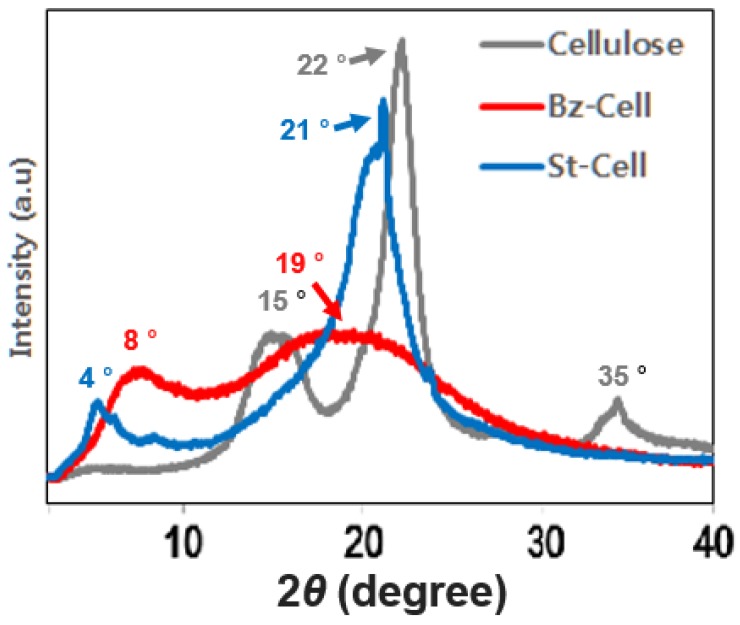
X-ray diffraction analysis (XRD) analysis of Cellulose (gray); Bz-Cell (red); and St-Cell (blue).

The influence of the crystalline behavior of pure cellulose and the modified celluloses of the esterification reaction on the crystallinity of cellulose were studied by wide angle XRD. Figure 5 shows the XRD diagrams of pure cellulose, the Bz–Cell, and the St–Cell. The conventional characteristic diffraction peaks of pure cellulose micro-crystalline were appeared at 2θ around 15°, 22°, and 35°. These characteristic peaks were changed after modification. Especially, the diffraction peaks of the Bz–Cell were showed a broader and lower intensity peak than those of pure cellulose because the benzoyl group could increase the amorphous character in the Bz–Cell. In the case of St–Cell, diffraction peaks, which were found at 4° and 21°, were different from the peaks of existing pure cellulose. However, a peak similar to pure cellulose was observed at 21°, rather than Bz–Cell, confirming that the stearoyl group had a relatively less effect on crystallinity change of pure cellulose [12,15,18]. From the above results, it was confirmed that crystallinity of the cellulose was changed by the application of benzoyl group and stearoyl group.

### 3.5. FE-SEM Analysis

**Figure 6:**
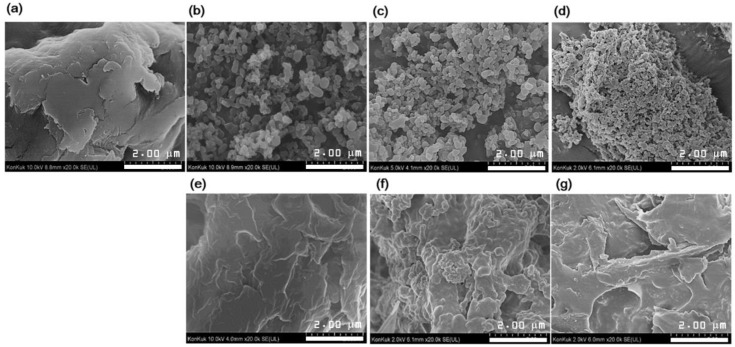
SEM images of cellulose (**a**); Bz–Cell (**b**); Bz–Cell after adsorption with phenanthrene (**c**); Bz–Cell after adsorption with pyrene (**d**); St–Cell (**e**); St–Cell after adsorption with phenanthrene (**f**); and St-Cell after adsorption with pyrene (**g**).

FE-SEM was used to study the morphological characteristics of the inclusion complex. The SEM images of cellulose, Bz–Cell, and St–Cell are shown in Figure 6a,b,e. Cellulose appeared as an irregular fold-like structure (Figure 6a), Bz–Cell as aggregated particles (Figure 6b) and St–Cell showed relatively cellulose-like surface morphology, but it is more rough, which is favorable for the adsorption of chemicals (Figure 6e) [28]. These surface phenomena correspond to the XRD results. We also analyzed the surface morphology of the modified cellulose after PAHs adsorption. After phenanthrene adsorption onto the Bz–Cell (Figure 6c), the materials appeared as more aggregated small particles because the benzoyl groups on Bz–Cell could interact with the poly aromatic rings of phenanthrene by π–π interactions. This tendency was also confirmed to the other Bz–cell after the adsorption with the pyrene with a larger poly aromatic rings than phenanthrene (Figure 6d). Figure 6f,g show the morphological surface characteristics of the St–Cell after adsorption with phenanthrene and pyrene, respectively. Differences in the flatness of the material were clearly observed before and after the adsorption of the phenanthrene and pyrene on the St–Cell. The difference of surface roughness was also shown in the FE-SEM data. However, St–Cell did not show any aggregations even in the presence of PAHs. We expect that the aggregation appeared in the Bz–cell might be a disadvantage in adsorbing PAHs because of the reduced surface area. In the case of the St–Cell shown in Figure 6f,g, both phenanthrene and pyrene were well attached on its surface, since the aliphatic groups on St–Cell could interact with the aromatic rings on PAHs.

### 3.6. Emission Spectra and Removal Test

**Figure 7:**
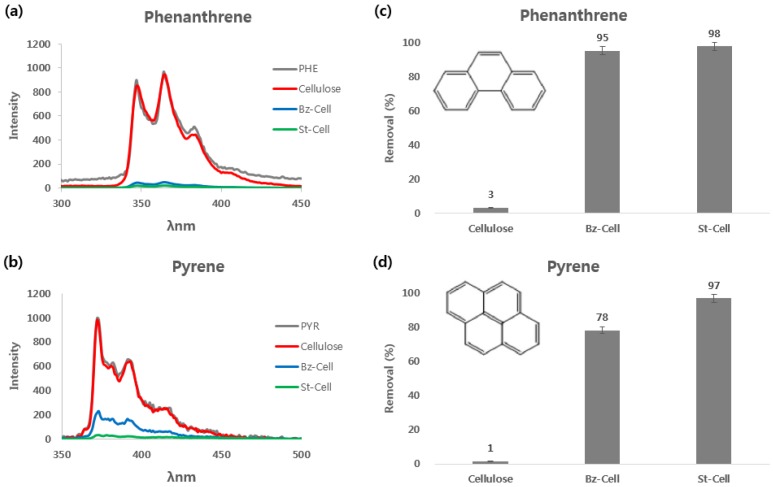
Emission spectra of phenanthrene (**a**) and pyrene (**b**). Removal efficiency (%) of phenanthrene (**c**) and pyrene (**d**), as compared with cellulose. The insets in (**c**) and (**d**) show the chemical structure of phenanthrene and pyrene, respectively.

**Figure 8:**
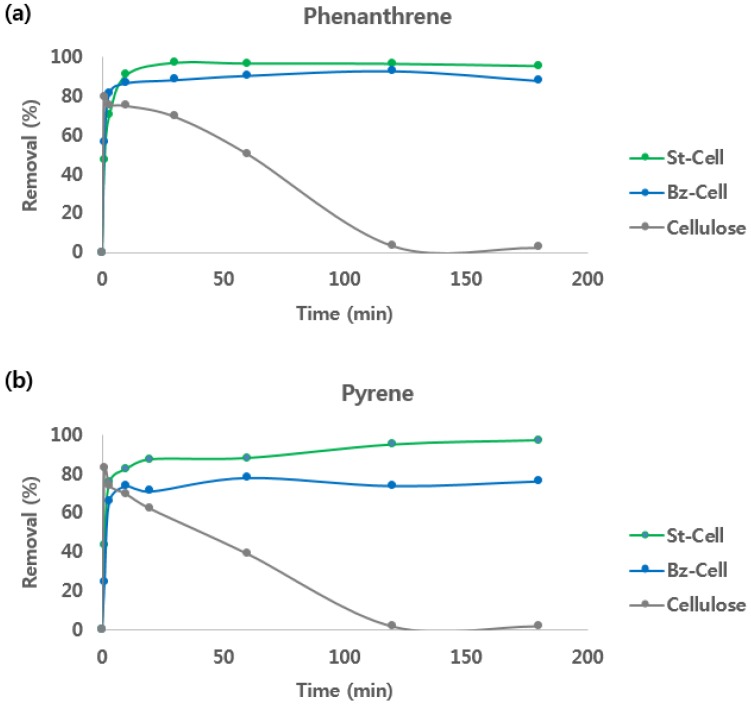
The time-dependent removal (%) of phenanthrene (**a**) and pyrene (**b**) by the Bz–Cell and St–Cell, compared with cellulose.

To clarify the sorption effects of PAHs onto the Bz–Cell and St–Cell, the fluorescence emission spectra of the PAHs were measured in the phenanthrene and pyrene solution of 0.8 and 0.08 ppm. After 24 h of stirring to equilibrate between the adsorbents and PAHs in aqueous solution, the emission spectra of the remained PAHs in solution were measured for the analysis. Each experiment was triplicated to obtain mean results (percentage removal of PAHs) [29]. The intensity of phenanthrene was not significantly affected by the addition of cellulose, while the addition of the Bz–Cell and St–Cell decreased the concentration by 95% and 97%, respectively (Figure 7a,c). In the case of pyrene, cellulose also had no effect, while the Bz–Cell and St–Cell displayed 78% and 96% removal efficiency (Figure 7b,d). Meanwhile, activated carbon and polystyrene are well known adsorbents for pollutants. Especially, the phenanathrene adsorption capacities of absorbent-grade activated carbon and polystyrene were around 8 to 50 and 3.91 mg/g, respectively [30,31]. In the case of polystyrene, the adsorption capacity was relatively small when compared to our material. Therefore, we tested the adsorption capacity of adsorption-grade activated carbon against PAHs. We used the Carbon black XC-72, which is commercially available adsorbent-grade activated carbon [32]. In the case of activated carbon, it showed the higher removal efficiency of 98% in phenanthrene and 99% in pyrene than those of Bz–Cell and St–Cell (Figure S3, Supplementary Materials). However, it showed very lower recovery efficiency of 8% in phenanthrene and 5% in pyrene. It also meant that the activated carbon could not be reusable practically due to its very low recovery. We actually measured the fluorescence of methanol from washing step of the activated carbon (Figure S1, Supplementary Materials). Those results indicate that reactivation of activated carbon is very difficult. It was also reported that activated carbons are expensive and their recovery are very difficult [2,33]. Efficient both recovery and recyclability with relatively high removal efficiency could be the strong advantages of our modified cellulose comparing with the activated carbon. The contact time significantly affected the adsorption of phenanthrene and pyrene. The effect of time on adsorption was determined by collecting the sample at pre-determined time intervals, up to the 180 min stirring time. The adsorption capacity of the prepared Bz–Cell and St–Cell showed rapid adsorption up to almost 10 min; as the adsorbent was mixed up to 10 min stirring time, the adsorption capacity gradually increased, and at 10 min, the adsorbent reached the saturation point. After this, no major adsorption changes were found. The time-dependence curve (0–180 min) of phenanthrene and pyrene adsorption is shown in Figure 8. The expected equilibrium point is at 10 min for both materials in a phenanthrene or pyrene solution of 0.8 and 0.08 ppm, respectively. This result can be explained by the materials’ hydrophobicity. By contrast, pure cellulose is a hydrophilic material. Cellulose is easy to disperse in water to bring the solid surface close to phenanthrene and pyrene in water. However, modified cellulose has organic chains grafted onto the surface, which enhance the interaction with phenanthrene and pyrene molecules, resulting in a higher adsorption capacity than pure cellulose.

### 3.7. Reusability of Bz–Cell and St–Cell

**Figure 9:**
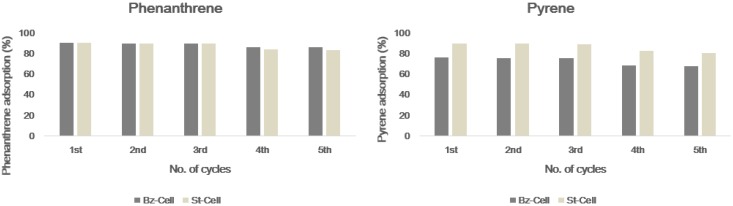
Recycling of Bz–Cell and St–Cell for the removal of phenanthrene and pyrene.

Finally, the regeneration and reusability of the Bz–Cell and St–Cell for the sorption of the model PAHs were examined. Figure 9 shows the adsorption percentage of the model PAHs after consecutive recycling of the Bz–Cell and St–Cell. The removal efficiencies of phenanthrene in the Bz–Cell and St–Cell remained above 83.9% for up to five sequential cycles. Both adsorbents still have enough efficiency to be reused even after five cycles. However, some reduction of recyclability were observed at the 10th cycle. We expect to use it up to 15 cycles with more than 50% efficiency (Figure S2, Supplementary Materials). Thus, these modified celluloses could be used repeatedly as efficient adsorbents to remove phenanthrene and pyrene from aqueous solutions.

## 4. Conclusions

Cellulose materials, modified by benzoyl and stearoyl groups, were prepared by a simple chemical grafting method. Using these reactions, functionalized cellulose, the Bz–Cell and St–Cell respectively, was successfully synthesized. The characterization results that were obtained by NMR, FT-IR, TGA, XRD, and SEM indicated that the benzoyl and stearoyl groups were grafted on the cellulose backbone. The Bz–Cell and St–Cell were estimated to have ability of potential adsorbents to remove two model of PAHs (phenanthrene and pyrene) in adsorption study. Especially, both the removal and reusability efficiency of St–Cell are higher than those of Bz–Cell, as shown in Figure 7 and Figure 9. This might be due to the aggregated surface properties of Bz–Cell with benzoyl moieties as shown in Figure 6b. In addition to the comparison between the two modification groups, both modified cellulose adsorbents showed higher adsorption efficiencies than previous studies [24]. Although the activated carbon showed the high PAHs absorptions, it has a clear disadvantage of very low recovery. However, the Bz–Cell and St–Cell can show the excellent both recovery and reusability with relatively high adsorption percentages of phenanthrene above 83.9%, up to the fifth cycle. Furthermore, the cellulose polymer is cheap comparing with activated carbon. Cellulose microcrystalline (powder, 20 µm Sigma-Aldrich) costs $150 per kilogram, as compared to $1000 for activated carbon Carbon black XC-72 (Fuel Cell Earth). This study could suggest that further research into the use of cellulose with various functional substituents can be applied to the molecular adsorbent of various organic pollutants in industrial scales, as well as for the removal of PAH. We also expect that the Bz–Cell and St–Cell would have the potential as basic biomaterials for various formulation studies, such as hydrogels, membranes, and filters.

## Figures and Tables

**Table 1:** FT-IR absorption bands for cellulose, Bz–Cell, and St–Cell.

Assignment	Cellulose (cm^−1^)	Bz–Cell (cm^−1^)	St–Cell (cm^−1^)
ν (O–H)	3331.4	-	-
ν (C–H)	2897.6	2907.6	2917.8
ν (C=O)	-	1723.1	1743.3
ν (C–O–C)	1028.6	1062.6	1052.9
ν (C–C(=O)–O–C)	-	1220.0	1233.2
C=C aromatic ring	-	1642.0	-

**Table 2:** TGA data of cellulose, Bz–Cell, and St–Cell.

Sample	Initial Decomposition Temperature, *T*_i_ (°C)	Glass Transition Temperature, *T*_g_ (°C)	50% Weight Loss Temperature (°C)
Cellulose	200	328	364
Bz–Cell	278	379	385
St–Cell	268	341	350

## Supplementary Materials

The following are available online at http://www.mdpi.com/2073-4360/10/9/1042/s1, Figure S1: Recovery percentage (%) of PAHs from Bz-Cell, St-Cell, and activated carbon by MeOH, Figure S2: Recycling of Bz-Cell and St-Cell for the removal of phenanthrene and pyrene up to 10th cycles, Figure S3: Removal efficiency (%) of phenanthrene and pyrene by activated carbon.
