# Supplementary material for: Efficient Adsorption on Benzoyl and Stearoyl Cellulose to Remove Phenanthrene and Pyrene from Aqueous Solution
Source: Polymers (Basel). 2018 Sep 19;10(9):1042. doi: 10.3390/polym10091042 (PMC6403814; doi:10.3390/polym10091042)
Supplement: Supplementary file 1 [file polymers-10-01042-s001.pdf]

polymers

MDPI

Supplementary information

Efficient Adsorption on Benzoyl and Stearoyl Cellulose to Remove Phenanthrene and Pyrene from Aqueous Solution

Yohan Kim [1,†], Daham Jeong [1,†], Kyeong Hui Park [1], Jae-Hyuk Yu [2] and Seunho Jung [1,*]

[1] Department of Systems Biotechnology, Microbial Carbohydrate Resource Bank (MCRB), Center for Biotechnology Research in UBITA (CBRU), Konkuk University, Seoul 05029, Korea; shsks1@hanmail.net (Y.K); amir@konkuk.ac.kr (D.J); kyeonghee17@naver.com (K.H.P)
[2] Departments of Bacteriology and Genetics, University of Wisconsin-Madison, Madison, WI 53706, USA; jyu@wisc.edu
* Correspondence: shjung@konkuk.ac.kr; Tel.: +82-2-450-3520
† These authors contributed equally to this work.

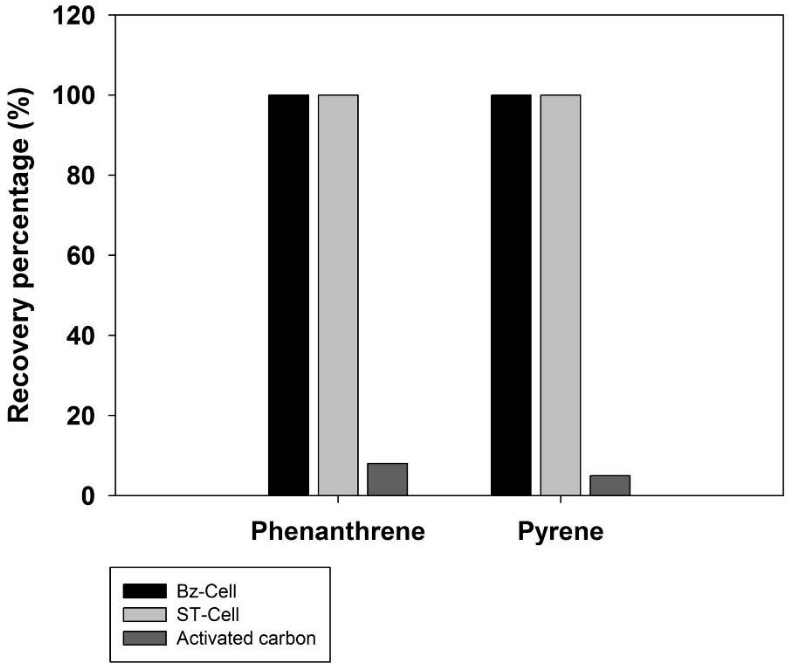

Figure S1. Recovery percentage (%) of PAHs from Bz–Cell, St–Cell, and activated carbon by MeOH.

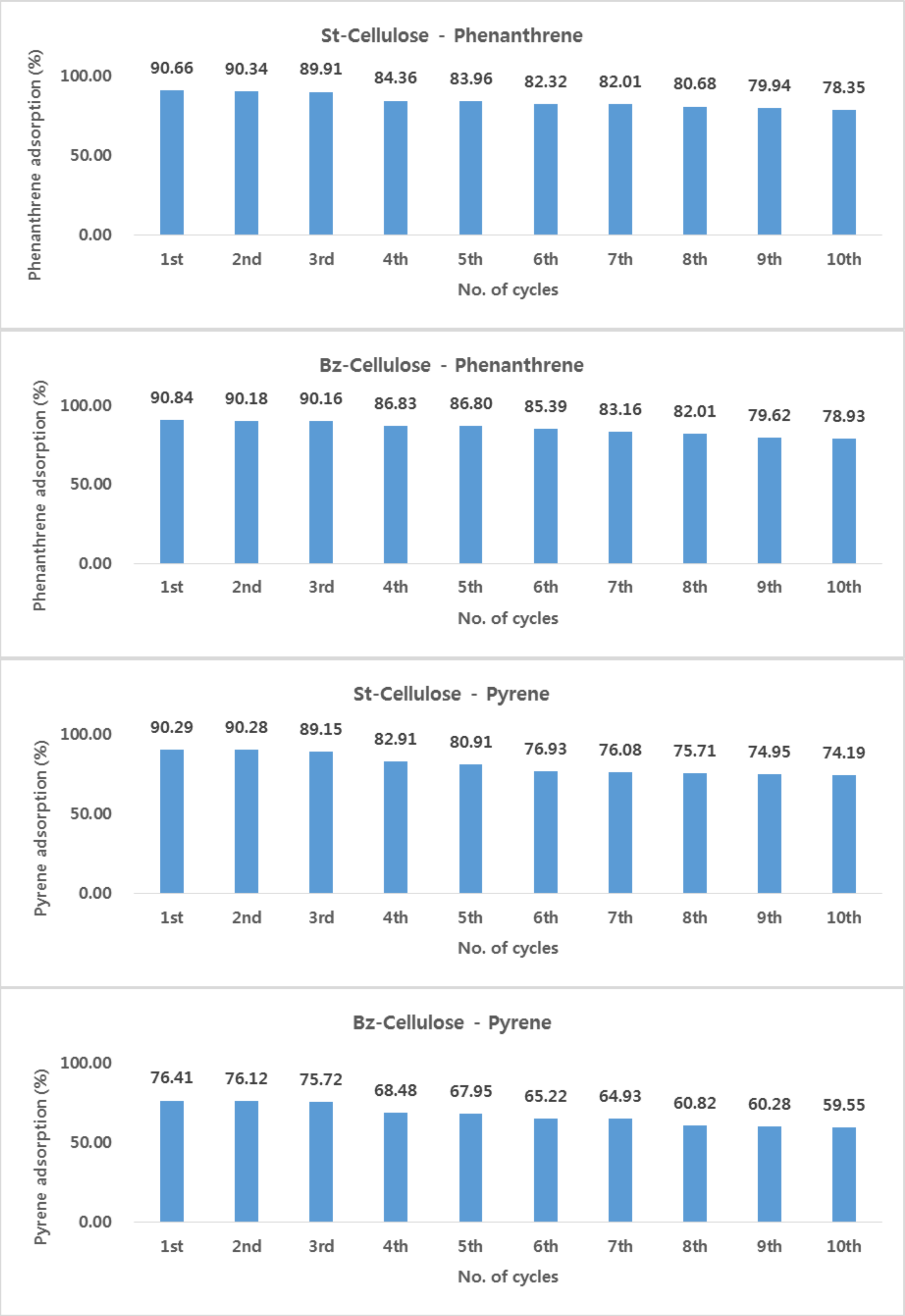

Figure S2. Recycling of Bz–Cell and St–Cell for the removal of phenanthrene and pyrene up to 10th cycles.

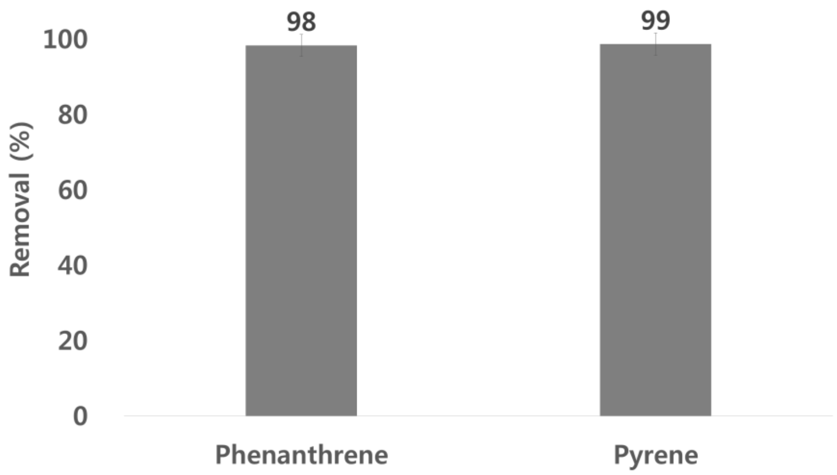

Figure S3. Removal efficiency (%) of phenanthrene and pyrene by activated carbon.
